# Host-directed immune therapies: a second front in the battle against sepsis

**DOI:** 10.1172/JCI204736

**Published:** 2026-06-15

**Authors:** Richard S. Hotchkiss, Guillaume Monneret

**Affiliations:** 1 Department of Anesthesiology, Washington University in St. Louis, St. Louis, Missouri, USA.; 2 Hospices Civils de Lyon, Immunology Laboratory, Hôpital E. Herriot, Lyon, France.

## Abstract

Each year, sepsis claims more lives in the United States than many major cancers and HIV/AIDS combined, yet therapeutic progress has been modest. Adding to this crisis is the alarming rise of multidrug-resistant “superbugs,” which increasingly render conventional antibiotics ineffective. Pathogen-targeted antibiotics will always remain a cornerstone of sepsis treatment, and research into novel antibiotics must continue unabated. However, the consistent mortality in sepsis tells us this approach is insufficient. Most deaths in sepsis do not occur during the early cytokine storm–driven hyper-inflammatory phase but rather days or weeks after the initial insult, during a protracted phase of immune suppression. Here, we make the case that a crucial way to reduce sepsis mortality lies in restoration of the patient’s immune competence, enabling the patient to contain and kill the invading microbes. Adjuvant immune therapies will not only enable killing of the initial, invading pathogens but also prevent secondary, hospital-acquired infections. Immunotherapy revolutionized oncology by challenging the assumption that cancer was best treated through cytotoxic or targeted tumor-directed approaches, and sepsis now stands at a similar inflection point. We argue that embracing immune restoration as a core therapeutic objective offers the most promising means to improve survival in this lethal disorder.

## Introduction

Sepsis is a life-threatening syndrome in which a dysregulated host response to infection leads to acute organ dysfunction, characterized by simultaneous hyper-inflammation and profound immune suppression (1). Sepsis remains one of the most lethal and least effectively treated syndromes in modern medicine (1–4). The prevailing theory of sepsis for many decades was that death was due to cytokine storm leading to refractory shock and multiorgan failure (5–8). Indeed, there are patients with sepsis who succumb to refractory shock and organ injury due to uncontrolled inflammation secondary to hyper-cytokinemia (Table 1) (9–11). Examples of these cases are patients with sepsis due to particularly virulent pathogens such as *Clostridia difficile*, *Neisseria meningitidis*, and group A β hemolytic *Streptococcus*.

However, more recent studies indicate that sepsis immunopathophysiology is more complex (12, 13). In the comprehensive REALISM study, investigators examined circulating cytokines serially in patients with sepsis and compared results with healthy volunteers, patients who had major surgery, and patients who sustained severe trauma (13). As a group, patients with sepsis had higher levels of circulating pro-inflammatory cytokines in the initial 24–48 hours of their illness compared with trauma or postsurgical patients. However, by 72 hours, the levels of cytokines in septic patients were comparable to trauma patients and to patients who underwent major surgery. Bauer et al. reported a similar marked decrease in levels of circulating cytokines in septic patients occurring by day 3 (14). Although the efficacy of antibiotics in rapidly reducing bacterial load partly contributes to this decline in cytokines, these findings, also observed to a lesser extent in sterile injuries, suggest that the initial hyper-inflammatory phase of sepsis is short-lived, consistent with the clinical course in which patients often present with fulminant, vasopressor-dependent shock. Vasoplegic shock and myocardial suppression are mediated by pro-inflammatory cytokines, and most patients with refractory septic shock tolerate reductions or discontinuation of vasopressors and ionotropic agents by days 2–3 of their illness. Improved clinical management has resulted in most of these patients surviving this initial phase of the infection (3, 15). Exact numbers are complicated because many of the patients who succumb to early severe sepsis do so because family members decide to forgo aggressive care and to focus on patient comfort instead.

**Figure 1:**
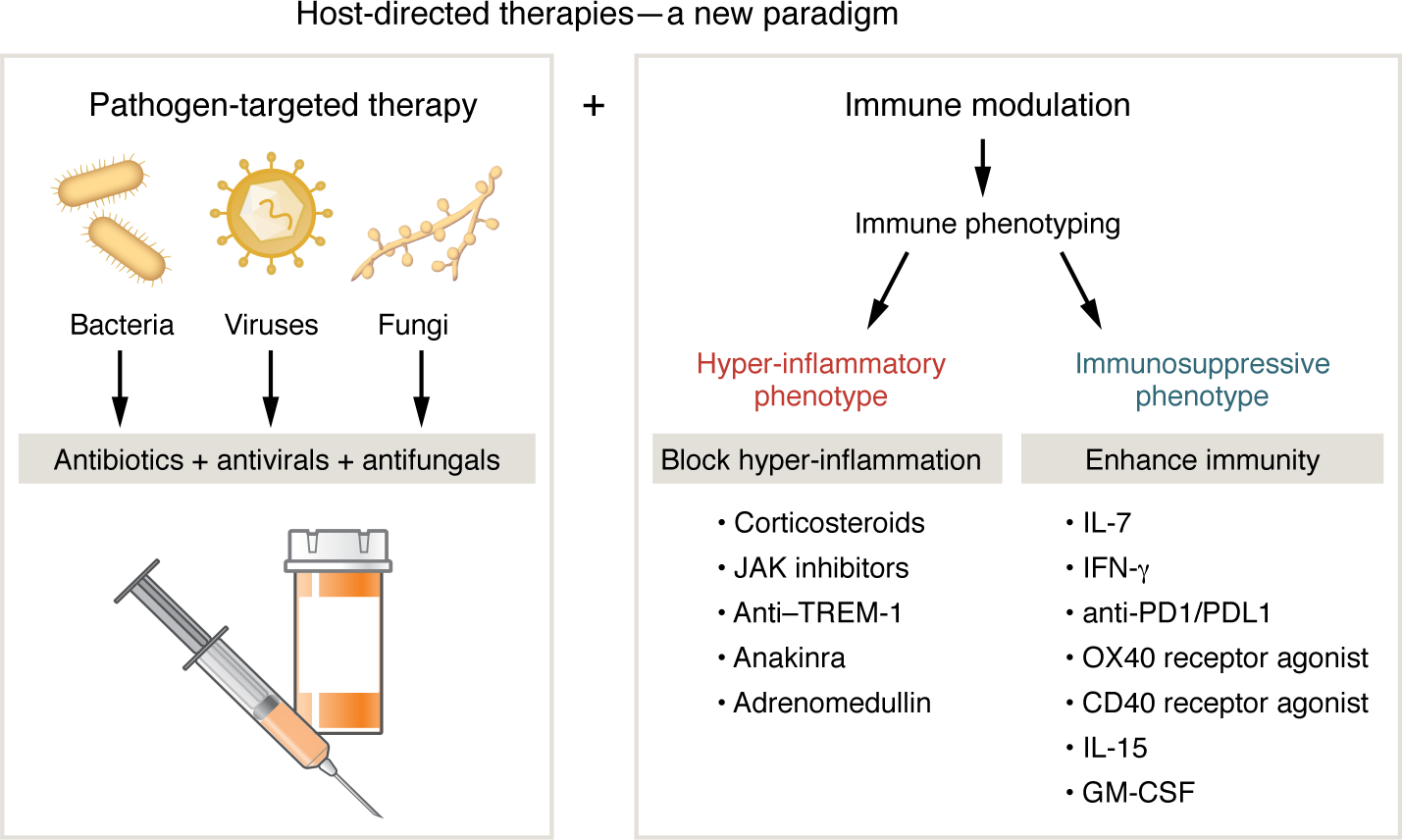
Host-directed therapies. In addition to antibiotics, immune modulatory therapies are likely to play an increasingly vital role in infectious diseases, including sepsis. Therapies to dampen an overexuberant hyper-inflammatory response or to boost an inadequate immune response will be selected based upon immune phenotyping. Immune adjuvant therapies to enhance host immunity will be important because most deaths from sepsis occur in patients with impaired immunity due to age (immunosenescence) or comorbidities. Immune adjuvant therapies offer key advantages in that they are pathogen agnostic (protect against a broad range of microorganisms, including multidrug-resistant pathogens) and will not induce antibiotic resistance.

Further complicating the picture, the extent and duration of the early inflammatory immune response are highly variable and affected by many factors, including size of the bacterial inoculum, type of pathogen, patient age, nutritional status, comorbidities, and genetic factors. For instance, up to 20% of patients who present with sepsis have hypothermia, evidence of failure to mount an effective inflammatory response (16, 17). Their elevated mortality underscores the danger of further suppressing immune function in individuals who are already failing to mount an adequate inflammatory response. Here, we argue for a new therapeutic paradigm that focuses on host-directed therapies in addition to traditional treatment as a second highly promising front in the war against sepsis (Figure 1).

## Immunosuppression as a key driver of sepsis mortality

The preponderance of evidence supports the conclusion that immunosuppression is the driving force behind morbidity and mortality in sepsis in patients who survive the initial hyper-inflammatory phase (Table 2). The demographic pattern of sepsis mortality provides one of the clearest indicators that immune failure lies at the heart of the sepsis syndrome. Most patients dying of sepsis are elderly or have comorbidities, such as malignancy, diabetes, or chronic renal failure, that compromise host defense mechanisms (18, 19). This pattern is difficult to reconcile with a purely hyper-inflammatory model but aligns precisely with a syndrome characterized by profound immune dysfunction.

**Figure 2:**
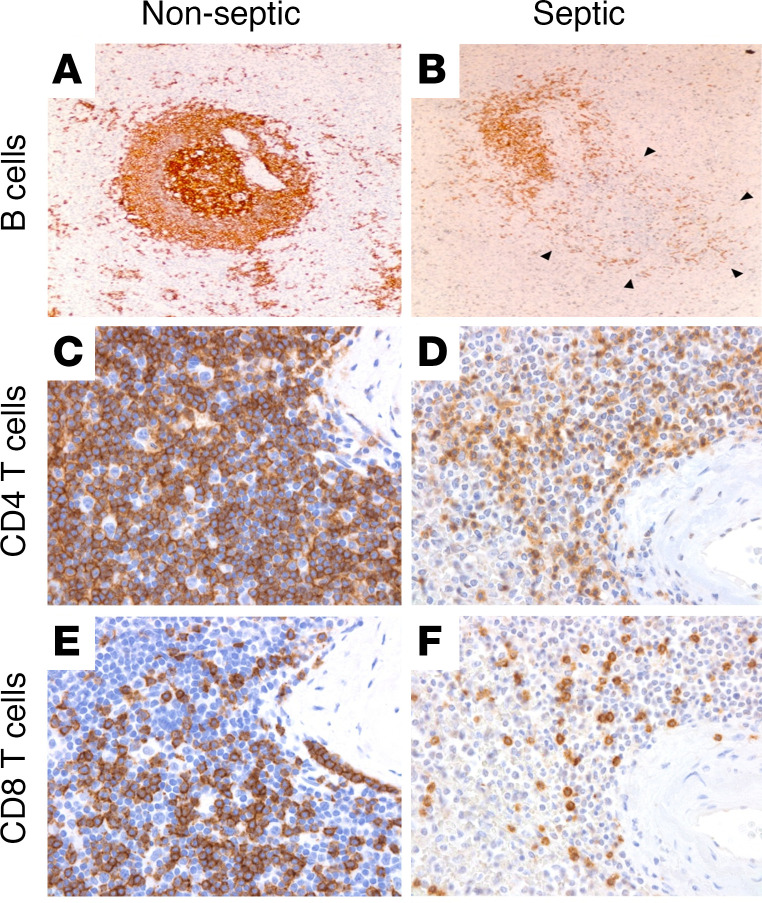
IHC staining of spleens from nonseptic or septic patients showing profound loss of immune effector cells in sepsis. IHC staining for B cells (CD20) in a lymphoid follicle (200× original magnification) of a nonseptic patient (**A**) and septic patient (**B**) showing loss of B cells in a septic patient; arrowheads indicate outline of lymphoid follicle with extensive loss of cells. IHC staining of CD4^+^ T cells in nonseptic (**C**) and septic (**D**) spleens showing severe CD4 depletion in sepsis. IHC staining for CD8^+^ T cells (400× original magnification) in nonseptic (**E**) and septic (**F**) spleens showing massive loss in sepsis. Panels **A** and **B** reproduced with permission from *The Journal of Immunology* (23). Panels **C**– **F** reproduced with permission from *JAMA* (21).

**Figure 3:**
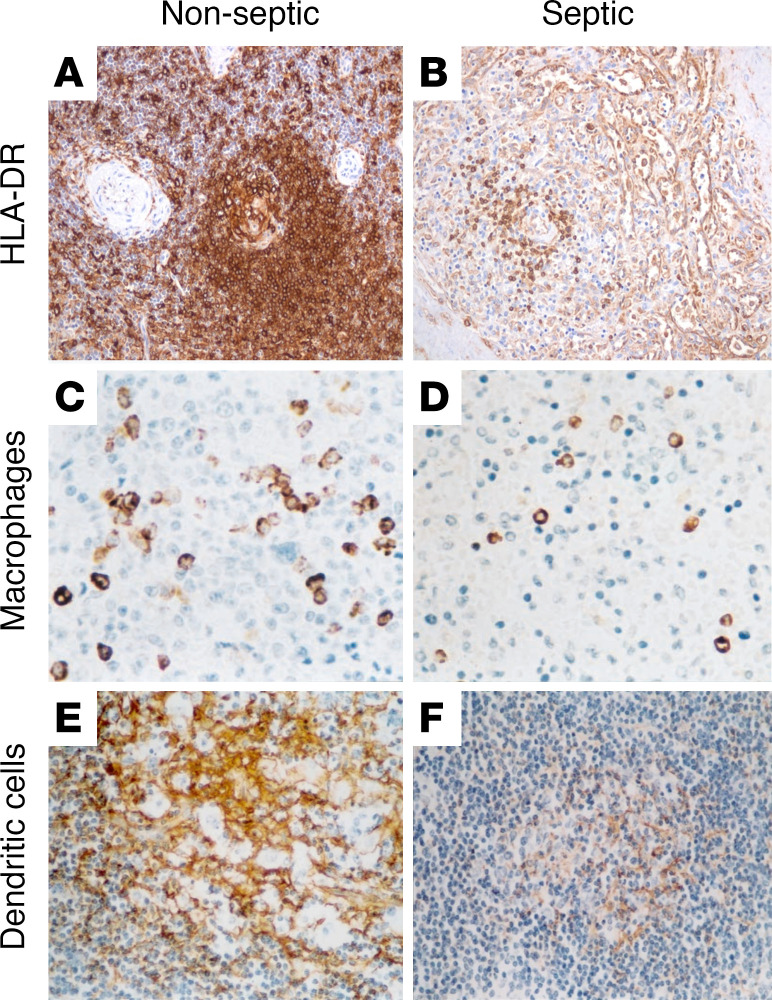
IHC staining of spleens from nonseptic or septic patients showing decrease in HLA-DR expression and loss of DCs but not macrophages in sepsis. IHC staining (200× original magnification) for HLA-DR in spleen from nonseptic (**A**) and septic patient (**B**). **A** shows robust periarteriolar staining in T and B cell zones consistent with major histocompatibility (MHC) class II pattern. **B** shows marked loss of HLA-DR reactivity in B and T cell zones typical of nearly subtotal depletion of HLA-DR–reactive elements in sepsis. IHC staining for macrophages (CD14) showing no differences in the number of splenic macrophages in nonseptic (**C**) versus septic spleen (**D**). (**E** and **F**) IHC staining for DCs (CD21) (400× original magnification). Note the profound loss of DCs in the septic versus nonseptic patient. Panels **A** and **B** are reproduced with permission from *JAMA* (21). Panels **C**–**F** are reproduced with permission from *The Journal of Immunology* (24).

Postmortem analyses of adult and pediatric patients dying from sepsis consistently reveal a striking apoptotic depletion of CD4^+^ and CD8^+^ T cells, B cells, and dendritic cells (DCs) in lymphoid organs and the spleen (Figures 2 and 3) (20–23). Sepsis-induced innate immune dysfunction is highlighted by a remarkable decrease in splenic monocyte and DC numbers and HLA-DR expression (Figure 3) (24), consistent with profound systemic immune collapse. Functionally, this depletion erodes both antigen presentation and effector T cell responses. The simultaneous loss of multiple immune cell lineages in patients dying of sepsis highlights a remarkable degree of immunosuppression, leaving patients unable to eradicate residual pathogens.

Reactivation of latent viruses in sepsis provides additional evidence of immune exhaustion. In healthy individuals, latent viruses such as CMV, EBV, and herpes simplex virus are kept in check by continuous immune surveillance. In septic patients, these viruses can reactivate, a phenomenon observed in more than 50% of individuals with prolonged critical illness (25, 26). Viral reactivation, as confirmed by detection of viral DNA in seropositive sepsis patients, correlates with longer intensive care unit (ICU) stays, higher rates of secondary bacterial and fungal infections, and increased mortality (25, 26).

Approximately 30%–50% of patients surviving the initial septic insult subsequently develop new infections with opportunistic pathogens, such as *Candida*, *Acinetobacter*, *Stenotrophomonas*, or *Pseudomonas* (27, 28). These pathogens rarely cause disease in immunocompetent hosts but readily occur in the immunosuppressed milieu of sepsis. Postmortem examination of surgical ICU patients who were admitted with a diagnosis of sepsis showed a continuous septic focus in approximately 77% of the autopsies (28, 29). These residual abscesses or micro-colonies of bacteria indicate that the host’s immune system failed to sterilize infected tissues despite antimicrobial therapy, a hallmark of impaired host immunity.

The importance of immune suppression in sepsis mortality is also made apparent in the outcomes in the approximately 10%–20% of patients whose initial presentation is characterized by hypothermia (16, 17). Patients who fail to mount a febrile response during sepsis have the highest mortality. These patients exhibit diminished pro-inflammatory cytokine production when their blood is stimulated ex vivo (30). Thus, the absence of inflammation in this context signals not homeostasis but the collapse of host defense mechanisms.

The link between cytokine production and mortality further implicates impaired immune function in sepsis. In ex vivo studies of blood from patients with sepsis, patients who had the lowest production of IFN-γ and TNF-α had the highest mortality, while patients who had the highest production of these pro-inflammatory cytokines had the lowest mortality (31, 32). In addition, a decreased capacity to release IFN-γ in whole blood assays was independently associated with clinical deterioration, including the occurrence of secondary infections or death (33).

Finally, perhaps the most compelling evidence for impaired immunity in sepsis is provided by studies examining the loss of the DTH response (34). The DTH test is a classic test of cellular immunity that evaluates the response (induration) to common recall antigens that are injected intradermally. Meakins and colleagues reported that surgical ICU patients who had an impaired DTH response, including many who were septic, had over 70% mortality versus an approximate 5% mortality in patients who had an intact DTH response (34). Although DTH testing is no longer routine in ICUs, the findings remain a vivid demonstration of the degree of functional immune failure in sepsis.

Collectively, these diverse lines of evidence from pathology, immunophenotyping, cytokine production, latent viral reactivation, and susceptibility to opportunistic infections point toward persistent immunosuppression as a central pathophysiologic feature of late sepsis. Indeed, the profound immune collapse that occurs during the late phase of sepsis is biologically equivalent to a state of acquired immunodeficiency. Recognizing this provides the biological foundation for immune adjuvant therapies aimed at restoring host defense rather than suppressing inflammation.

## Mechanisms of sepsis-induced innate and adaptive immune suppression

Sepsis induces myriad defects in innate and adaptive immunity, but due to space constraints we discuss only several key immune-suppressive mechanisms. Readers are referred to several excellent reviews on this topic (2, 4, 5, 35, 36).

### Rapid shift of myeloid cells toward immunosuppressive functions.

Upon infection, innate immune cells are rapidly activated. These cells swiftly recognize pathogens through the activation of pattern recognition receptors, triggering the release of inflammatory cytokines and chemokines. The rapid increase in circulating cytokines in septic patients causes a shift in the phenotype of myeloid cells. Inflammatory signals cause the bone marrow to release immature neutrophils (emergency hematopoiesis), which progressively evolve toward myeloid-derived suppressor cells (MDSCs) and suppress T cell activation and proliferation. Neutrophils and macrophages become less effective at engulfing and destroying pathogens (37, 38), and DCs, monocytes, and macrophages downregulate key surface molecules like HLA-DR and costimulatory molecules, leading to impaired antigen presentation (39–42). Overall, there is a shift from a pro-inflammatory cytokine profile (e.g., TNF-α, IFN-γ, IL-1β, IL-18) to an antiinflammatory or immune-suppressive profile (e.g., IL-10, TGF-β). This imbalance makes it difficult for patients to eradicate their primary infection and renders them more susceptible to secondary, hospital-acquired infections.

### Apoptosis-induced depletion of immune cells.

An additional cause of immunosuppression in sepsis is apoptosis-induced loss of lymphocytes. The loss of lymphocytes occurs systemically in lymphoid and nonlymphoid organs alike, including spleen, lung, and gastrointestinal tract (20–24). Autopsy samples from spleens of patients with sepsis showed a remarkable depletion in CD4^+^ and CD8^+^ T cells, B cells, and DCs compared with splenic samples from patients with nonseptic etiologies (Figures 2 and 3) (21–24). This massive loss of lymphocytes is documented in adult, pediatric, and neonatal patients dying of sepsis and occurs in sepsis due to Gram-negative, Gram-positive, and fungal pathogens (20, 22, 43). The decrease in tissue lymphocytes is paralleled by a decrease in circulating lymphocytes (lymphopenia), one of the most common laboratory findings in patients with sepsis. Importantly, numerous studies have reported that the severity of lymphopenia correlates with sepsis outcomes (20, 44, 45). Moreover, independent investigators have shown that blocking sepsis-induced apoptosis using an array of antiapoptotic therapies improves survival in clinically relevant animal models of bacterial and fungal sepsis (46–48). These studies suggest that the loss of immune effector cells is a critical pathogenic mechanism in sepsis.

In contrast with the profound apoptosis of effector lymphocytes, there is a rise of suppressive lymphocytic lineages including Tregs during sepsis (49). This is most notably reflected in the decreased production of IFN-γ, a cytokine central to host defense during sepsis (50). In terms of prognostic significance, defects in IFN-γ release are more predictive than lymphocyte counts. IFN-γ production is reduced by 50%–70% in splenocytes from patients dying of sepsis compared with splenocytes from patients who died of nonseptic causes (21, 32, 33, 51, 52). Additionally, the capacity for lymphocyte proliferation is decreased after sepsis, whether in response to recall antigens or mitogens (49). Loss of cytokine production and reduced proliferation are hallmark features of lymphocyte exhaustion. This is further corroborated by the increased expression of the negative co-stimulatory molecule programmed cell death 1 (PD-1), a marker of T cell exhaustion, on circulating lymphocytes in septic patients (53). Notably, anti–PD-1 antibodies can restore T cell IFN-γ production in these patients (54) as discussed below.

### Persistent inflammation, immunosuppression, and catabolism syndrome.

Despite the overall suppression of critical cellular elements of innate and adaptive immunity during prolonged sepsis, damaging inflammation and muscle protein breakdown (cachexia) persists, a condition termed persistent inflammation, immunosuppression, and catabolism syndrome (PICS) (55). Persistent, low-grade fevers, along with elevated C-reactive protein (CRP) and other acute-phase proteins, reflect ongoing cytokine-driven inflammation. This can be a particularly prolonged problem, continuing even after resolution of the acute phase of sepsis and leading to ongoing tissue damage, organ dysfunction, and delayed recovery. As previously discussed, this promotes differentiation of MDSCs rather than neutrophils, which increases susceptibility to secondary infections (37). Additional features of PICS include severe metabolic imbalances, excessive protein breakdown, cachexia, and impaired tissue repair. The crosstalk between the inflammatory cascade and the coagulation and complement systems likely also contributes to sustained inflammation through positive feedback mechanisms (2). It is likely that multiple cell types contribute to this low-grade inflammation, including activated vascular endothelial cells, various parenchymal cells, and subtypes of monocytes, macrophages and neutrophils from damaged organs. Overall, most patients with sepsis become progressively immunosuppressed, often to a profound degree, while presenting signs of persistent inflammation. Understanding the vicious cycle linking low-grade inflammation and immunosuppression is of the utmost importance.

## Calming the storm: the antiinflammatory strategy

### Old molecules, novel trial designs.

There is a strong rationale for using antiinflammatory treatments in the very first hours of sepsis onset or ICU admission, yet many of the antiinflammatory drugs that have been tested in randomized controlled trials (RCTs) have shown little to no success, including corticosteroids, tocilizumab (anti–IL-6), anakinra (recombinant IL-1 receptor antagonist), eritoran (anti-TLR4), and drotrecogin alfa (activated protein C) (56, 57). Several excellent reviews have examined the topic of corticosteroids in sepsis (58–60). Intriguingly, new evidence suggests that low-dose corticosteroids have effects to enhance adaptive immunity (61, 62). One widely accepted explanation for their failure in sepsis is the lack of consideration for the heterogeneity of patients’ inflammatory profiles. As previously described, in the first few days after ICU admission, some patients exhibit extreme inflammation, while others have already spontaneously controlled their inflammatory response (7, 8, 11, 12). Therefore, administering antiinflammatory treatment to all patients without first assessing the extent of their inflammatory response has likely played a large role in the unsuccessful, and sometimes harmful, outcomes of most studies (63–65). Accordingly, the current shift is toward so-called “enrichment strategies” based on inflammatory biomarkers and clinical characteristics to identify individuals for whom the chances of therapeutic success with a given molecule are higher (63).

In consideration of this change, several teams have performed post hoc analyses on antiinflammatory RCTs. IL-1 blockade (anakinra) was associated with a marked improvement in survival of patients with sepsis and macrophage activation syndrome versus patients receiving placebo (64, 65). Anakinra also reduced adjusted mortality from 45% to 34% in individuals with high inflammatory markers, whereas mortality in individuals with lower inflammatory profiles was unaffected (64). More recently, many studies assessed anti–IL-6 or anti–IL-1 therapeutic strategies in COVID-19 patients without stratification and provided both positive and negative results depending on centers and the types of cohorts (66, 67). In contrast, when IL-6 was retrospectively assessed, early IL-6 blockade (tocilizumab) was associated with improvement and increased survival in patients with high levels of IL-6 (i.e., >30 pg/mL). Patients with high circulating IL-6 levels treated with placebo as well as patients with low circulating IL-6 levels treated with tocilizumab showed the highest mortality (67).

Using a transcriptomic approach, Antcliffe and colleagues retrospectively observed that different endotypes of septic patients, as defined by mRNA signatures, responded differently to steroids (68). Patients were subphenotyped through latent class analysis (LCA), which integrates numerous clinical parameters and routinely available biomarkers, to identify two distinct patient phenotypes: hyper-inflammatory and hypo-inflammatory. Notably, steroids were effective only in the hyper-inflammatory group. Furthermore, a reanalysis of drotrecogin alfa’s effect in adults with septic shock (PROWESS-SHOCK study) using LCA revealed that the drug improved survival in the hyper-inflammatory subgroup but worsened outcomes in the hypo-inflammatory subgroup (69).

Although few, prospective studies incorporating patient enrichment in their design should be highlighted. For instance, in severe pneumonia, identified by physiological instability and laboratory abnormalities, corticosteroids reduced disease progression in patients with CRP levels above 150 mg/L (70, 71). Importantly, the validity of this approach was demonstrated during the COVID-19 pandemic. A prospective RCT of anakinra, using suPAR (a soluble inflammatory biomarker) as a stratification marker, showed clinical benefits in severe COVID-19 patients with elevated suPAR concentrations (72). In a (non–COVID-19) randomized, precision-immunotherapy trial conducted in 276 patients with sepsis across six countries, patients were stratified based on immune status into two groups: those with hyper-inflammation due to macrophage activation-like syndrome (MAS), defined by elevated ferritin levels, and those with immunoparalysis, characterized by low ferritin and decreased monocyte HLA-DR (mHLA-DR) expression (73). Patients with MAS were treated with anakinra while patients with immunoparalysis were treated with IFN-γ. A control group received placebo. Although there was no difference in 28-day mortality, patients who received precision-guided immunotherapy had improved organ failure scores compared with patients receiving placebo. These studies support the adoption of a guided, individualized treatment strategy over a one-size-fits-all approach.

### Newer immunosuppressive drugs.

In addition, some new approaches to downmodulate the inflammatory pathway in sepsis are showing promising preliminary results (Figure 1 and Table 3). The ASTONISH trial was a phase IIb randomized, double-blind, placebo-controlled study designed to assess the efficacy and safety of nangibotide in 355 patients with septic shock. Nangibotide is a novel therapeutic agent that inhibits the TREM-1 signaling pathway, which plays an important role in inflammation loops during sepsis (74). The study was conducted in patients with elevated soluble TREM-1 (sTREM-1) levels as an inclusion parameter within the first 24 hours after admission. Treatment was stopped when the patients were weaned off catecholamines. While the study did not meet its predefined objective (change in sequential organ failure assessment [SOFA] score by day 5) in patients with sTREM-1 ≥ 656 pg/mL, it provided critical data on the design and feasibility of an upcoming phase III trial.

JAK inhibitors are used extensively in patients with autoimmune diseases to block inflammation by inhibiting the inflammatory cascade (75). Several large trials of JAK inhibitors were conducted in COVID-19, with some studies showing beneficial effects while other studies showed lack of efficacy (75, 76). The differing findings were likely due to differences in trial design and the severity of illness of the patient populations. Baricitinib, an inhibitor of JAK-1 and JAK-2, was the first immunomodulatory treatment to receive FDA approval for treating COVID-19 among hospitalized adults requiring respiratory support. Based upon this, baricitinib and other JAK inhibitors are now being assessed in multiple platform trials in sepsis and ARDS (NCT06381661, ISRCTN82395639, and ISRCTN81435672). The potential benefits of JAK inhibitors in sepsis are likely to be dependent upon early administration in patients experiencing an excessive hyper-inflammatory response. These adaptive platform trials incorporate both biological baselines and clinical and biological responses to treatment for inclusion and continuation and are expected to provide valuable insights into the personalization of sepsis therapies.

A key hallmark of septic shock is increased vascular permeability with resultant loss of intravascular volume and accompanying peripheral edema (2, 3, 77). Adrenomedullin is essential for maintaining endothelial barrier health and function (77) and inhibits pro-inflammatory cytokine production by monocytes and macrophages. Preclinical animal models of sepsis demonstrated that increasing circulating adrenomedullin improved endothelial homeostasis, organ function, and survival (77). A clinical trial of a high-affinity, non-neutralizing anti-adrenomedullin antibody that acted to increase the circulating half-life of the active moiety of adrenomedullin (to prolong its protective effect on endothelial cells) was safe and well tolerated in 301 patients with septic shock (77). Treatment was also associated with improved ΔSOFA scores, consistent with a beneficial effect. A larger phase III trial is planned.

### Clinical considerations for use of immune-suppressive drugs.

Collectively, there is strong rationale for antiinflammatory drug strategies in sepsis, although it is likely to apply only to a minority of patients. Indeed, although no prospective data are available, the proportion of patients affected by unbridled inflammation is estimated to be between 12% and 22% (78–80). In addition to restricting this therapy to patients with cytokine-driven hyper-inflammation (see Table 1 for clinical characteristics), several other key principles are essential for successful trials of antiinflammatory, immune-modulatory drug trials in sepsis. These include: (a) early administration (within the first 24–48 hours) during the initial hyper-inflammatory phase; (b) restriction to drugs with short half-lives to avoid exacerbating the subsequent immunosuppressive phase; and (c) selection of a specific antiinflammatory therapy based upon immune markers, such as ferritin, CRP, and IL-6 levels. While these questions fall outside the scope of this Review, they clearly warrant in-depth discussions among experts and intensivists.

## Activating the troops: immune adjuvant therapy

The immunosuppressive phase of sepsis may persist for several days to many months, as evidenced by an increased incidence of secondary hospital-acquired infections, viral reactivation, prolonged ICU stays, and the fact that infections remain the leading cause of readmission and mortality after surviving an initial sepsis episode (81). Indeed, the immunosuppressive phase is believed to account for up to 70% of the total sepsis-related mortality (9, 28, 78–80). Importantly, as advances in intensive care have improved survival during the initial hyper-inflammatory phase, the number of patients transitioning into the immunosuppressive phase grows. As a result, restoring immune function has emerged as a key factor in improving survival. This hypothesis is reinforced by the transformative success of immunotherapy in oncology, as cancer and sepsis share similarities in immune dysfunction (82–84). To date, several drugs that boost host immunity have shown promising results in animal models of sepsis, case reports, and small clinical trials (Figure 1 and Table 3). A few phase II RCTs have been conducted, yielding encouraging results. Despite hypothetical concerns about reactivating a hyper-inflammatory response, no safety issues have yet emerged when trials identified the most immunosuppressed patients based on biomarkers. Concerns and possible adverse effects of immune adjuvant therapies in sepsis are described in relevant sections below.

### IFN-γ.

Human recombinant IFN-γ-1b is a small molecule classified as an immunostimulant cytokine and immunomodulator. Briefly, IFN-γ polarizes macrophages into the pro-inflammatory (M1) phenotype, enhancing their microbial killing ability, phagocytosis, and inflammatory cytokine release while upregulating MHC class II (MHC-II) molecules (39, 85). Additionally, IFN-γ promotes Th1 polarization in T cells and enhances CD8^+^ T cell cytotoxic activity. Recombinant IFN-γ-1b has been a therapeutic option for 30 years, approved in Europe (under the names IMMUKIN and IMMUKINE), Australia, and the United States (as ACTIMMUNE) for reducing the frequency of serious infections in patients with chronic granulomatous disease or severe malignant osteopetrosis and as an adjuvant with other therapies (39, 85). RCTs of IFN-γ treatment have yielded underwhelming yet interesting results in sepsis (86–88). Pioneering, though nonblinded, work in this field involved the use of IFN-γ in patients with low mHLA-DR expression, demonstrating efficacy in restoring immune function and reducing mortality (39). Another small RCT, also based on low mHLA-DR levels, reported a reduction in pulmonary nosocomial infections in trauma patients treated with IFN-γ (89). Several IFN-γ–based RCTs are currently underway or planned; many of these studies are guided by low mHLA-DR expression, including the INFINITY (NCT06694740), IGNORANT (NCT05843786), IMMUNOSEP (NCT04990232), and PLATINUM (NCT06774235) trials. Underscoring the complexity of the sepsis field, the EMBRACE trial (NCT06694701) is evaluating therapy with emapalumab, a monoclonal antibody that blocks IFN-γ signaling, in patients who are thought to be in an IFN-γ–mediated hyper-inflammatory phase of sepsis (90).

*GM-CSF: a two-edged sword?* GM-CSF is currently used to accelerate myeloid cell recovery in the setting of bone marrow suppression after hematopoietic stem cell transplantation. The rationale for use of GM-CSF in sepsis lies in its ability to upregulate MHC-II and costimulatory molecules on monocytes, macrophages, and DCs, along with increasing the production of TNF-α, IL-6, and IL-12. In a prospective trial including 38 patients with severe sepsis or septic shock, biomarker-guided GM-CSF therapy (i.e., mHLA-DR < 8,000 antibodies per cell) was safe and effective in restoring monocytic immunocompetence (91). A study based on low ex vivo TNF release in children with organ failure demonstrated a strong protective effect of GM-CSF against nosocomial infections (40). A recent RCT evaluating GM-CSF in patients stratified by low mHLA-DR failed to show efficacy in preventing ICU-acquired infections, but the study was underpowered due to premature termination (92). GM-CSF is considered a double-edged sword in cancer immunotherapy, as it may indirectly promote expression of the inhibitory receptor PD-1 on T cells (92–94). Additionally, GM-CSF may induce release of immunosuppressive MDSCs (94). Its assessment in sepsis, including close monitoring of immune function, warrants further investigation in larger patient cohorts.

### Immune checkpoint inhibitors: anti–PD-1 and anti–PD-L1.

Based on the success of immune checkpoint inhibitors in cancer, it has been hypothesized that blockade of the PD-1/PD-L1 axis could ameliorate sepsis-induced immunosuppression by restoring T cell function. Sepsis animal models showed that anti–PD-1 and anti–PD-L1 antibodies enhanced immunity and improved survival (54, 95). Additionally, lymphocytes from septic patients treated with anti–PD-1/PD-L1 antibodies demonstrated improved proliferation and cytokine production (54, 95, 96). Phase I/II studies with nivolumab (anti–PD-1) and anti–PD-L1 (BMS-936559) have reported acceptable safety profiles without evidence of inducing cytokine storm or autoimmunity (97, 98). These studies also showed biological signals of restored immune function, including increased monocytic HLA-DR expression and improved T cell activity (97, 98). Nivolumab demonstrated a half-life of approximately 15 days and a sustained receptor occupancy of more than 80% for up to 28 days. These findings suggest that a single dose of nivolumab could be sufficient to treat most septic patients, contrasting markedly with oncology protocols, where repeated cumulative dosing contributes to the emergence of adverse effects. Furthermore, in silico modeling studies indicate that the dose of nivolumab necessary in sepsis could be substantially lower than in oncology, decreasing the risk of inducing severe autoimmunity (99).

Invasive fungal infections typically occur in patients with impaired immunity, including trauma and cancer patients, often presenting with increased T cell expression of PD-1 (100, 101). Although no clinical trials of checkpoint inhibitors have been conducted in the setting of fungal infections, murine studies demonstrated that PD-1/PD-L1 inhibition enhanced T cell effector function, increased cytokine release, and improved survival (86, 96, 100, 101). Case reports also describe trauma or oncology patients who developed refractory invasive fungal infections despite maximal antifungal therapy and were subsequently treated with anti–PD-1 therapy, either alone or in combination with IFN-γ (86, 102–104). In these reports, immune checkpoint inhibition with nivolumab was associated with improved control of invasive fungal disease with recovery.

Importantly, additional studies are necessary to thoroughly evaluate safety concerns for potential use of checkpoint inhibitors in patients with sepsis. All checkpoint inhibitors have the potential to cause serious autoimmune side effects by blocking essential inhibitory molecules that restrain autoreactive T cells. The incidence of immune-related adverse events (hepatitis, colitis, pneumonitis, and thyroiditis) in oncology patients treated with checkpoint inhibitors is approximately 10%–30% (105). These autoimmune episodes can be life-threatening and have even been reported after a single dose of checkpoint inhibitors. These events frequently require treatment with high-dose corticosteroids or other immunosuppressants, which could worsen the underlying condition of patients with sepsis. Thus, careful consideration as to the risk/benefit ratio of administering anti–PD-1/PD-L1 antibodies to septic patients is necessary, and only patients with refractory sepsis are likely good candidates for this type of immune adjuvant therapy.

### IL-7: master regulator of immunity.

**Figure 4:**
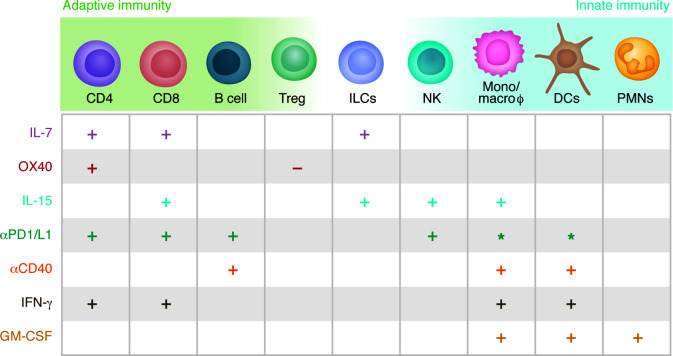
Host-directed therapies targeting adaptive and innate immunity. Specific immune adjuvant drug therapies target cells comprising innate and adaptive immunity. Treg, T regulatory cell; ILCs, innate lymphoid cells; DCs, dendritic cells; PMNs, polymorphonuclear leukocytes; *,checkpoint inhibitor therapies may have additional effects through retrograde signaling on monocyte/macrophages and/or dendritic cells, though the impact of these therapies on these cell types is not well characterized.

IL-7 has been called the “maestro of the immune system” because of its effects on diverse immune cells involved in pathogen elimination (Figures 1 and 4 and Table 4) (106). IL-7 is the most effective cytokine for expanding and sustaining CD4^+^ Th cells, which are profoundly depleted in sepsis (Figure 2) (47). In septic patients, loss of CD4^+^ and CD8^+^ T cells leads to impaired pathogen clearance and vulnerability to secondary bacterial and fungal infections (107). IL-7 reverses these defects by preventing lymphocyte apoptotic cell death and by increasing lymphocyte proliferation (108–111). IL-7 thus effectively reverses T cell exhaustion and restores production of IFN-γ, which stimulates monocytes and macrophages to phagocytose and kill invading pathogens (21, 108, 112, 113). IL-7 induces production of IL-17, causing neutrophil mobilization and migration to sites of infection (112). Furthermore, IL-7 activates mucosal-associated invariant T cells that line the gastrointestinal and respiratory tracts, which prevent invasion by microbial organisms (114, 115).

Another potential benefit of IL-7 is its effect to upregulate expression of lymphocyte adhesion molecules, resulting in increased lymphocyte trafficking to abscesses or poorly perfused sites of infection (47, 115). Additionally, IL-7 induces the production of stem-like CD4^+^ and CD8^+^ T cells that ensure a continuous release of effector T cells that are essential for long-term immunity in chronic infection (116). Finally, IL-7 activates resident memory T cells to respond rapidly upon pathogen reexposure at barrier sites (114).

Over 600 adult and pediatric severely lymphopenic patients with varying presentations have been treated with IL-7; underlying causes include refractory infection, sepsis, and cancer treatments in clinical trials (110, 115). Septic patients with lymphopenia treated with IL-7 reacted with a 3- and 4-fold increase in circulating CD4^+^ and CD8^+^ T cells (110). Importantly, the increase in lymphocytes was long-lived, persisted for months after cessation of IL-7 therapy, and potentially decreased the incidence of new secondary, hospital-acquired infections and/or reduced hospital readmissions for recurrent sepsis.

In a recent double-blind, randomized, placebo-controlled trial of IL-7 in 109 critically ill patients with COVID-19, IL-7 was safe and well tolerated, the only exception being a single occurrence of transient respiratory deterioration (117). Patients treated with IL-7 had a 44% decrease in nosocomial infections and a decrease in length of ICU stay (117). These clinical results support the contention that IL-7 can safely boost immune recovery in critically ill, lymphopenic patients. Notably, IL-7 cannot be given intravenously because its unique receptor-mediated clearance mechanism results in circulating levels 100 times higher than when it is delivered subcutaneously or intramuscularly, which can drive transient reversible respiratory distress (111).

### IL-15: a pleiotropic cytokine.

IL-15, like IL-7, is a member of the common γ-chain family of cytokines. IL-15 promotes the survival, proliferation, and activation of CD8^+^ T cells, NK cells, monocyte/macrophages, and DCs (118). Several key differences in IL-7 and IL-15 should be noted. Unlike IL-7, IL-15 does not act upon CD4^+^ Th cells. In contrast, IL-15, but not IL-7, directly activates monocyte/macrophages, which express the IL-15 receptor complex. Thus, IL-15 has direct effects on both adaptive and innate immune cells (Figure 4). In two clinically relevant animal models of sepsis, CLP and *Pseudomonas aeruginosa* pneumonia, IL-15 improved survival and ameliorated sepsis-induced loss of CD8^+^ T, NK, and dendritic cells (119). Currently, there are no previous or ongoing clinical trials of IL-15 in sepsis or other infectious diseases. However, IL-15 is in multiple early-phase clinical trials in oncology and was recently approved clinically for treatment of bladder cancer, in which IL-15 is administered by bladder irrigation. Results of early oncology trials show that IL-15 expands NK and CD8^+^ T cells without inducing a dose-limiting cytokine storm when given subcutaneously at low concentrations (120). IL-15’s effect to expand memory/effector CD8^+^ T cells, even in the absence of strong T cell receptor stimulation, may be especially advantageous in septic patients given their frequent depletion of DCs and low HLA-DR expression (Figure 3). However, IL-15’s effect to trigger T cell receptor–independent stimulation could worsen sepsis-induced immunopathogenesis (118). IL-15 is more likely than IL-7 to exacerbate sepsis-induced hyper-inflammation (121). While some sepsis animal models show that IL-15 improves survival, others have shown that IL-15 may heighten inflammation and worsen sepsis mortality (119, 121). Thus, careful dose titration and timing of administration will be imperative in any future sepsis trials. Intriguingly, IL-15 may be particularly efficacious in the treatment of viral and intracellular bacterial pathogens because it activates NK cells and CD8^+^ cytotoxic T cells, which play a key role in eliminating intracellular pathogens (118). We also speculate that administration of IL-15 by inhalation rather than by subcutaneous route could lead to local control of respiratory viruses with reduced risk of inducing excessive systemic inflammation. Results of the ongoing clinical trials of IL-15 in oncology patients will provide guidance on its potential clinical utility in infectious diseases.

### Agonistic antibody to CD40: an activator of innate immunity.

While IL-7, and to a lesser degree IL-15, are highly effective at activating adaptive immunity, for host-directed therapy to be optimally effective, immune adjuvants that reverse sepsis-induced suppression of the innate immune system are also needed. A similar search for immune drug therapies that activate innate immunity is underway in oncology. One approach is the use of agonistic antibodies to the CD40 receptor, which is expressed on DCs, monocyte/macrophages, and B cells (Figure 4) (122). These agonistic antibodies are highly potent activators of DCs and enhance expression of MHC-II and CD80/CD86. In addition, CD40 agonistic antibodies increase DC production of IL-12, which drives an IFN-γ–mediated Th1 response, and induce B cell proliferation and antibody class switching to potentially improve pathogen clearance (122). Although first-generation agonistic antibodies to the CD40 receptor had adverse side effects, including cytokine release syndrome, thromboembolic events, and hepatotoxicity, newer generation Fc-engineered antibodies are much better tolerated (123). The results of these newer generation anti-CD40 receptor antibodies, e.g., mitazalimab (Alligator Bioscience), sotigalimab (Apexigen, Merck), and selicrelumab (Roche), have been encouraging, with some studies showing increased progression-free survival and occasional complete response (123).

Although there is a relative paucity of preclinical studies of agonistic antibody to the CD40 receptor in infectious models, some studies have shown efficacy in viral, parasitic, and bacterial sepsis models (124). Research from our laboratory using a mouse model of sepsis showed that agonistic antibody to CD40 ameliorated B and T cell death and improved survival in a two-hit model of CLP followed by bacterial pneumonia (125).

### OX40 receptor agonists: potent activators of T cells.

Although anti–PD-1 and anti–PD-L1 antibodies are highly effective in reversing T cell exhaustion and improving outcomes in patients with cancer, they only work in a subset of patients. Consequently, investigators have been searching for additional immune adjuvants that can restore or enhance CD4^+^ and CD8^+^ T cell responses. OX40 receptor, a costimulatory molecule present on CD4^+^ and CD8^+^ T cells, boosts T cell effector function and inhibits Tregs (Figure 4) (126–128). OX40 receptor agonists have undergone clinical trials in oncology patients with modest beneficial effects in a subset of patients (128). While the antitumor effects of OX40 receptor agonists are not as pronounced as anti–PD-1 antibodies, they have a more favorable safety profile without any confirmed cases of severe autoimmune side effects (126–128).

OX40 receptor agonist antibodies have shown beneficial effects in animal models of hepatitis B virus and in a Leishmania parasite model (129, 130). Studies by our group showed that agonistic anti-OX40 receptor antibodies ameliorated the sepsis-induced loss of CD4^+^ and CD8^+^ T cells, markedly enhanced IFN-γ production, and improved survival in a mouse CLP model of sepsis (131). Importantly, ex vivo stimulation of blood with agonistic anti-OX40 antibody was very effective in restoring lymphocyte IFN-γ production in patients with sepsis due to multidrug-resistant bacteria (132).

### Limitations of animal models and phase II sepsis trials.

It is important to underscore the limitations of using results from animal models of sepsis and small phase II trials to identify potential therapies. Widely used preclinical models, e.g., the CLP model, do not fully recapitulate the temporal complexity, comorbidities, and immunologic diversity occurring in patients. Most phase II clinical studies to date have been small, been nonrandomized, and been performed without biomarker-guided stratification. Future progress will depend upon rigorously designed, adequately powered trials that incorporate mechanistic biomarkers to align immunomodulatory therapies with patient-specific immune endotypes. Table 4 describes the characteristics of septic patients who may be the best candidates for immune adjuvant therapy.

## Immunotherapy: insights from oncology trials

One striking lesson of the last decade is that many cytokines do not behave in the rigid pro-inflammatory/antiinflammatory framework that dominated earlier sepsis thinking. For example, IL-6 has long been labeled a pro-inflammatory mediator and is often cited as a central driver of early septic shock. Yet multiple clinical studies in patients with cancer indicate that blocking IL-6 signaling can enhance antitumor immunity (133, 134). This counterintuitive effect appears to stem from the fact that chronic IL-6 exposure promotes T cell dysfunction, reduces antigen presentation, expands MDSCs, and increases Tregs (134). Thus, in some settings, IL-6 signaling dampens, rather than amplifies, effective host immunity. It is conceivable that sustained IL-6 signaling in late sepsis reinforces immunosuppressive pathways. Therefore, blocking IL-6 may improve host immunity in a subset of patients with sepsis.

Equally surprising are the effects of JAK/STAT inhibition. JAK inhibitors were developed as antiinflammatory drugs, and at high doses they clearly suppress cytokine signaling. However, emerging data indicate that low-dose JAK inhibition can enhance immune function and improve tumor killing in patients with cancer (135, 136). These findings have potentially important implications for sepsis, where persistent, low-grade cytokine signaling is a hallmark of the immunosuppressive phase.

IL-10, historically viewed as the prototypical antiinflammatory cytokine, also demonstrates remarkable duality. Although IL-10 unquestionably suppresses monocyte activation in acute inflammation, clinical trials in pancreatic cancer showed that IL-10 administration expanded CD8^+^ T cells, increased IFN-γ production, and remarkably improved tumor control in a subset of patients (137, 138). Rather than simply shutting down immune responses, IL-10 appears capable of rescuing exhausted T cells under certain metabolic conditions. In this regard, the authors reported that IL-10 added ex vivo to patient blood samples *increased* lymphocyte IFN-γ production in a large cohort of patients (139).

Although corticosteroids are traditionally viewed as broadly immunosuppressive, several studies suggest that low-dose or context-specific exposure may enhance host defenses (60, 61). Glucocorticoids can exert a selective immunostimulatory effect on B cells, promoting more effective antibody responses (61). Shen and colleagues demonstrated that glucocorticoids can augment IFN-γ–mediated upregulation of monocyte antigen-presenting molecules (140). Shimba et al. showed that while corticosteroids suppress innate immunity, low-dose corticosteroids enhance adaptive immunity (62). These observations reinforce the broader theme that immune-modulating therapies may behave in unexpected, even opposite, ways depending on dose, timing, and the underlying immune state of the patient.

These unexpected findings serve as a cautionary note for sepsis. Cytokines and JAK/STAT-targeting drugs cannot be assumed to have fixed biologic effects throughout the course of the illness. During early septic shock, IL-6 or JAK inhibition could be beneficial, whereas in late sepsis, when many patients exhibit profound immune exhaustion, the same pathways may contribute to suppress host defense. This reinforces the central theme of the present Review: therapies must be matched to the patient’s immune state, not applied uniformly based on historical categorizations of cytokines as pro- or antiinflammatory. Moreover, in the future, it will be critical to consider the high proportion of sepsis patients with preexisting immunosuppression, including recipients of solid-organ transplants and bone marrow transplants, and patients receiving high-dose corticosteroids, biologics, or JAK inhibitors. These patients represent a uniquely vulnerable population when they develop sepsis, as reviewed elsewhere (141, 142), and will require tailored therapies.

## Conclusion

The traditional view of sepsis as a uniformly hyper-inflammatory cytokine storm is no longer tenable. While a small minority of patients, often younger individuals with highly virulent, toxin-driven infections, do succumb rapidly from cytokine-mediated multiorgan failure, the vast majority of deaths in sepsis now occur days to weeks later during a state of profound and persistent immunosuppression. Precision antiinflammatory therapy may benefit patients with true hyper-inflammatory profiles when given early, but it does not address the dominant lethal mechanisms. The future of sepsis therapy therefore lies in host-directed immune adjuvant therapy. Agents such as IFN-γ, IL-7, IL-15, checkpoint inhibitors (anti–PD-1/PD-L1), OX40 receptor agonists, and agonistic antibodies to CD40 have already shown, in preclinical models, patient case reports, and/or early clinical studies, that they can reverse sepsis-induced immunosuppression and improve outcomes. Of these, IL-7 is the most promising because of its effects to restore the number and function of CD4^+^ and CD8^+^ T cells, which are massively depleted in sepsis and play key roles in orchestrating the global immune response to kill invading pathogens.

Finally, the striking immunologic parallels between sepsis and cancer offer an optimistic forecast. Just as immunotherapy transformed oncology by unleashing the patient’s own immune system against cancer, precision immune restoration promises to do the same against sepsis. Rapid point-of-care phenotyping combined with adaptive trial platforms will enable clinicians to deliver the right immune therapy, either suppressive or stimulatory, to the right patient at the right time. We believe that the next decade will likely bring therapies that can restore immune homeostasis, prevent secondary infections, shorten ICU stays, and reduce the long-term burden carried by millions of sepsis survivors. In this regard, host-directed immune adjuvant therapy does not merely represent an incremental advance, but rather it offers a fundamentally new therapeutic framework with the potential to alter the history of sepsis. The opportunity before us is profound, i.e., to move from reactive treatment to potential preemptive, proactive restoration of immunity, and in doing so, to shift the trajectory of this devastating disease toward survival and recovery. Louis Pasteur once said, “Messieurs, c’est les microbes qui auront le dernier mot. (Gentlemen, it is the microbes who will have the last word.)” We assert that immune adjuvant therapy is not an incremental advance; it is the long-overdue second front that can finally give the host, rather than the microbe, the last word.

## Conflict of interest

The authors have declared that no conflict of interest exists.

## Funding support

- NIH R35GM1269 to RSH.

## Figures and Tables

**Table 4:**
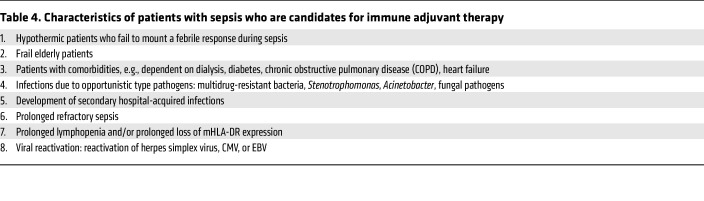
Characteristics of patients with sepsis who are candidates for immune adjuvant therapy

**Table 3:**
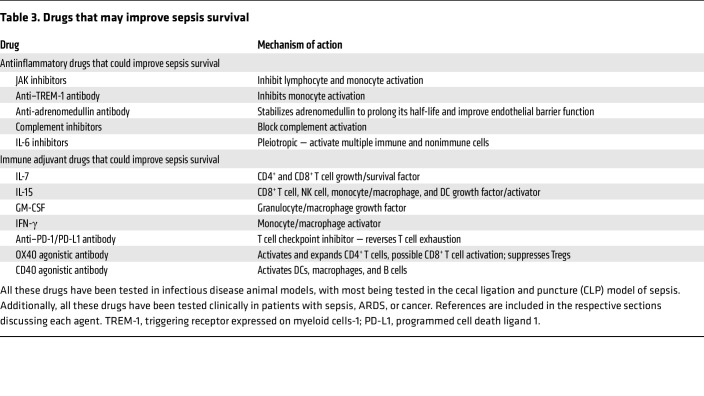
Drugs that may improve sepsis survival

**Table 2:**
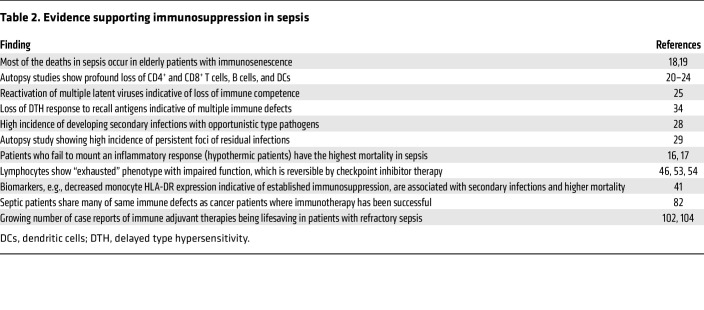
Evidence supporting immunosuppression in sepsis

**Table 1:**
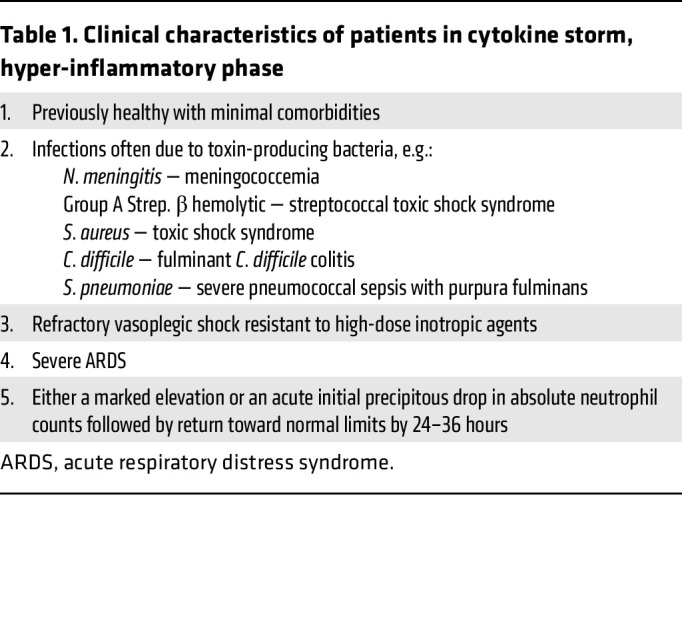
Clinical characteristics of patients in cytokine storm, hyper-inflammatory phase

## Version 1 06/15/2026

Electronic publication
